# Evaluation of temperature sum models and timing of *Quassia amara* (Simaroubaceae) wood-chip extract to control apple sawfly (*Hoplocampa testudinea* Klug) in Sweden

**DOI:** 10.1007/s10340-014-0616-0

**Published:** 2014-08-18

**Authors:** P. Sjöberg, W. Swiergiel, D. Neupane, E. Lennartsson, T. Thierfelder, M. Tasin, B. Rämert

**Affiliations:** 1 Department of Plant Protection Biology, Swedish University of Agricultural Sciences, P.O. Box 102, SE-230 53 Alnarp, Sweden; 2 Department of Energy and Technology, Swedish University of Agricultural Sciences, P.O. Box 7032, SE-750 07 Uppsala, Sweden

**Keywords:** IPM, Organic, Forecasting, Monitoring

## Abstract

Apple sawfly (*Hoplocampa testudinea* Klug) is a serious pest in European organic apple production. They hatch during a short period only, making correct timing of control measures crucial. Swedish organic growers have requested a strategy for optimal timing of the *Quassia amara* (Simaroubaceae) extract against the apple sawfly. The aim of this study was, therefore, to develop methods to predict the timing of *Q. amara* control in Sweden. A temperature sum model for timely placement of monitoring or mass-trapping sticky traps was validated for Swedish conditions. The average emergence of sawflies occurred at 169 degree days (SD = 20) counted from March 15 (threshold temperature 4 °C). The difference in emergence from existing first flight model of average and maximum 9 and 39 degree days (1 and 9 calendar days) was found acceptable. Accumulated oviposition of 85 % at full bloom (BBCH 65) suggests that mass trapping and monitoring could stop at this time. This is supported by a tendency of decreased trap catches during that period. Three application times for *Q. amara* were compared: (A) at petal fall (BBCH 67), (B) at a date calculated using female trap catch numbers and temperature sums, and (C) prior to peak egg hatch observed in the field. All treatments resulted in significantly lower percentage of damaged apples compared to the unsprayed control, with significantly less damage (1.3 %) in plots treated according to method (B). The results provide information on adult phenology and methods that could be used to determine timing of mass trapping and insecticide application against the apple sawfly.

## Key message

- Forecasting methods for pesticide application against the apple sawfly were validated for Swedish conditions by application of the bio-pesticide *Quassia amara*.
- Timing of intervention, based on a date calculated using female trap catch and temperature sums, achieved significantly less damage than models based on phenological plant stage or on observed peak of egg hatch.
- By applying *Q. amara* in accordance with a temperature model, a low impact strategy for the control of the sawfly in orchards is proposed.

## Introduction

Apple sawfly (*Hoplocampa testudinea* Klug; Hymenoptera: Tenthredinidae) is an important univoltine pest of apple in Europe and North America (Pyenson 1943; Vincent and Mailloux 1988; Cross et al. 1999; Velbinger 1939). The sawfly hibernates in the soil as pre-pupae and emerges in the flowering period of early and moderately early apple varieties (Graf et al. 2001; Ciglar and Baric 2002; Miles 1932). Accordingly, there is a link between the reproductive period of the apple sawfly and the phenological flowering stages of apple trees, which can be described by the biologische bundesanstalt, bundessortenamt und chemische industrie (BBCH) scale (Meier et al. 1994) and used to time control measures against the pest. Without pesticide application or parasitism, sawfly populations in the Netherlands can double every year (Zijp and Blommers 2002b). The damage caused by sawfly larvae comprises characteristic primary damage with winding tunnels under the fruitlet skin caused by 1st and 2nd instar larvae and secondary damage caused by older instars with direct entrance to the ovary and no winding scars (Miles 1932; Dicker and Briggs 1953). Apple sawfly control in Swedish conventional orchards relies on the use of non-selective synthetic insecticides (Manduric 2013), while control in European organic orchards commonly has relied on the use of a commercial or homemade extract from the shrub *Quassia amara* (Simaroubaceae) (Ascard and Juhlin 2011; Trapman pers. com. 2012). The short persistence of *Q. amara* extract avoids a negative effect on the later emerging apple sawfly parasitoid *Lathrolestes ensator* (Zijp and Blommers 2002a, b). The extract is sprayed after flowering to eradicate neonate larvae of the sawfly. Quassinoids such as quassin and neoquassin are reported to be the active toxins in *Q. amara* extract (Kienzle et al. 2006; Villalobos-Soto et al. 1999). The commercially recommended timing of *Q. amara* application, at petal fall, is estimated to coincide with egg hatch. Monitoring of adult sawfly emergence, estimation of population density, and mass trapping are possible using white sticky traps (Owens and Prokopy 1978; Wildbolz and Staub 1986; Graf et al. 1996a). The apple sawfly is visually attracted by the non-UV reflecting white color of the trap (Owens and Prokopy 1978). A majority of sawflies trapped are considered to have emerged from soil hibernation within the preceding 24 h (Graf et al. 1996b). The common recommendation is to place traps 10 days before bloom, since if they are installed too early they attract other insects and may lose their attractiveness (Graf et al. 1996c).

Monitoring with traps only provides an indication of the adult activity, without containing any information regarding the development of eggs and/or larvae. However, exact timing of spraying is an essential prerequisite for satisfactory control of apple sawfly (Höhn et al. 1993; Kienzle et al. 2002). As a consequence, several phenology-driven models based on soil and air temperature have been developed to predict the spring emergence of the adult sawflies, the time of mating, and the period of egg development (Graf et al. 1996a; Zijp and Blommers 1997; Graf et al. 2001, 2002). An early model employed soil temperature to estimate the emergence of adults (Graf et al. 1996a), but air temperature is cheaper and easier to measure and more commonly used by advisory services and growers for the management of other apple pest species (Jones et al. 2010; Roubal and Rouzet 2003; Agnello and Reissig 2010). A study by Zijp and Blommers (1997) showed that air temperature resulted in approximately the same precision as soil temperature. A better estimate of optimal insecticide timing can be obtained when the information from trap catches is combined with weather data and with empirically derived temperature sum for egg development (Graf et al. 2001, 2002). Soil texture has also been shown to influence the timing of sawfly emergence (Graf et al. 1996a) and this could theoretically alter the relationship between air and soil temperature, and hence the appropriateness of using air temperature at different locations. Furthermore, Graf et al. (1996b) found that the relationship between temperature and developmental stage of sawfly populations from different European regions differed significantly, even when the thermal threshold was similar. According to these authors, in order to expand the validity of the model, the temperature dependence of development has to be quantified for different populations.

The aims of the present study were to determine whether (*i*) the first trap catch of apple sawfly in Sweden could be predicted by the air-based temperature sum model of (Zijp and Blommers 1997) and used to indicate optimal timing of trap placement for monitoring or mass trapping; (*ii*) trap catches of sawflies decrease because of visual competition from the actual flowers during bloom; (*iii*) apple tree phenology would co-vary with observed egg and larval stages; and (*iv)* the efficacy of *Q. amara* treatment differs depending on time of application, e.g., at petal fall, or as recommended based on female trap catches and existing temperature sums for adult lifespan to egg development (Graf et al. 2001, 2002) or at observed egg hatch under Swedish conditions. Such information may be important when optimizing apple sawfly control measures within an integrated pest management approach.

## Material and methods

### Evaluation of first trap catch model

Populations of apple sawfly were monitored during 2011–2013 in seven orchards (1–7) in southern Sweden (Table 1). The orchards were located at least 20 km from each other and five were managed organically, while two were under integrated production (IP). During field seasons, weather data (air temperature, relative humidity, and rain) were recorded at each orchard using Vantage Pro weather stations (Davis Instruments, San Francisco, CA, USA). Rebell bianco sticky traps (Andermatt Biocontrol AG, Grossdietwil, Switzerland) were used to record flight pattern and monitor the emergence of adults. Six traps were placed in each orchard, at random positions approximately 1.5 m above the ground and at a minimum distance of 30 m from each other except in orchards one and three (minimum 10 m distance) due to smaller orchard size. Early flowering cultivars in Swedish organic orchards were used (Amorosa, Collina, Discovery, Holsteiner Cox, Nanna, Rubinola, Santana). The traps were placed in the orchards at the end of April and removed after the end of flight each year. They were replaced at each recording occasion. Trap catches at orchards one, two, and three were recorded every day due to the precision in timing required by the *Q. amara* trial. In the remaining orchards, trap catches were recorded twice a week to record the pattern of population emergence (Table 1). At each location, observed adult emergence was compared with the associated predicted emergence based on the temperature sum construct proposed by Zijp and Blommers (1997) (Table 2). The average temperature sum and standard deviation for all years and orchards were compared to the Zijp and Blommers (1997) reference value of 177 ± 10 degree days.

**Table 1:** Swedish apple orchards used to evaluate apple sawfly (*Hoplocampa testudinea*) phenology and/or management with *Quassia amara* extract (2011–2013)

Orchard	GPS coordinates (WGS84)	Size (ha)	Production system	Trap observation	Year	Experiment conducted
1	N 55° 43.229′, E 14°	0.25	IP	Every day	2011	Trap observation + *Q. amara*
					2012	Trap observation + *Q. amara*
					2013	Trap observation + *Q. amara*
2	N 56° 2.993′, E 12°	0.9	Organic	Every day	2011	Trap observation + *Q. amara*
					2012	Trap observation + *Q. amara*
					2013	Trap observation + *Q. amara*
3	N 56° 27.251′, E 12°	10	Organic	Every day	2013	Trap observation + *Q. amara*
4	N 55° 44.556′, E 13°	2	Organic	Twice per week	2011	Trap observation
					2012	Trap observation
					2013	Trap observation
5	N 55° 36.534′, E 14°	1.5	Organic	Twice per week	2011	Trap observation
					2012	Trap observation
					2013	Trap observation
6	N 55° 39.523′, E 14°	12	Organic	Twice per week	2012	Trap observation
					2013	Trap observation
7	N 56° 6.886′, E 14°	17	IP	Twice per week	2012	Trap observation

### Relationship between trap catches and flowering stages

Observed adult emergence was compared locally with apple tree phenology according to the BBCH scale (Meier et al. 1994).

### Relationship between tree phenology (BBCH), egg, and larval development

During 2013, egg and larval developments were compared with temperature sum forecasts from air temperature, trap catches, and apple tree phenology. The egg and larval observations were made every day and due to logistical and time restrictions the study was limited to orchards two and three. A minimum of 20 apples with either ovipositional scar, superficial tunneling or radial entrance holes were randomly collected from trees throughout each orchard. Their development stage was recorded by microscopy. Six egg stages, dsA-dsF, were determined from schematic drawings (Kuenen and Vrie 1951) and larval stage by measurement of head capsule size (Miles 1932). The temperature sum was obtained by entering recorded maximum and minimum daily air temperature data for each orchard into an Excel spreadsheet, and use pre-established formulae to calculate degree days (Snyder 1985).

### Application timing and efficacy of *Quassia amara*

A field trial with wood-chip extract of *Q. amara* was conducted in 2011 and 2012 at orchard one. The aim was to measure the efficacy of *Q. amara*, applied according to the common commercial practice of spraying at petal-fall (BBCH stage 67–69) (Treatment A), as compared with an unsprayed control. In 2013, the experiment was expanded to orchards two and three and included a study on the efficacy of *Q. amara* timed according to temperature sums (Treatment B). Temperature sums for adult lifespan, oviposition, and egg development proposed by Graf et al. (2001, 2002) were calculated, as described above, from daily recorded accumulated female trap catches and local weather data. The resulting accumulated curves indicated the theoretical egg hatch peak at which *Q. amara* application was performed. In a third treatment (Treatment C), based on field observations of egg development stages, *Q. amara* was sprayed when the majority of the eggs reached the final development stage F (dsF) before hatching. Treatment C was excluded from orchard one due to the low number of eggs found. Summary of treatments:

A: application at petal fall (BBCH stage 67–69 - year 2011–2013), orchards 1–3

B: application according to accumulated trap-catches + temperature sums (year 2013), orchards 1–3

C: application at final egg stage F, just prior to observed peak larvae hatching (year 2013), orchards 2–3

D: Control: unsprayed (year 2011–2013), orchards 1–3

*Quassia* extract applications were made according to a randomized complete block design, with four blocks per orchard. Every block contained all three treatments and the untreated control. The blocks contained 40 trees in a row and a group of 10 trees were randomly assigned to each treatment. A buffer zone between treatments was composed of one tree on each side. The effect of *Q. amara* was measured by counting the number of undamaged and damaged fruits on 3–5 randomly chosen trees in each block and treatment approximately 2 weeks after the first application and with the same trees sampled again after two additional weeks (damage check occasion 1 and 2, respectively, see Table 3 and Fig. 2). Fruit damage included primary and secondary damage caused by the apple sawfly (Dicker and Briggs 1953).

Wood chips were prepared according to the Swedish board of Agriculture recommendations of 12 kg/ha, with the chips infused in warm tap water (60 °C) for a period of 24 h and then filtered (Ascard and Juhlin 2011). The filtered extract was mixed with water and Zence 40 (potassium oleate) as a wetting agent (1 %) and application was carried out in late evening, using a back-pack sprayer (Solo Accu Power 416, Solo Kleinmotoren GmbH, Sindelfingen, Germany) at a field rate of 400 L/ha. High pressure liquid chromatography (HPLC) was used to determine the quassin content in the different wood chip batches in the experiment. The field dose of approximately 12 kg/ha of *Q. amara* was adjusted each year to correspond to 13.3 mg quassin/L. For the HPLC, an aliquot of 500 μL of the aqueous wood-chips extract was filtered in a 0.2 μm centrifugation filtration unit and centrifuged for 2 min (1,300 rpm) at room temperature. One hundred μL of the sample was injected into the HPLC. Each sample was injected twice. A Waters HPLC system consisting of two pumps and one automatic gradient controller was used. The detector was an Agilent 1,200 diode array detector working at 254 nm. The sample was introduced to the column via a Rheodyne injector fitted with a 100 μL loop. The HPLC-stationary phase consisted of a 4 µm C18 column (Waters, 3.9 × 150 mm) and the mobile phase was Milli-Q water with 1 % (w/w) acetic acid (pump A) and methanol with 1 % (w/w) acetic acid (pump B). Gradient separation was performed at a flow rate of 1.0 mL/min. The initial mobile phase ratio was 80 % A and 20 % B. A linear gradient to 40 % A and 60 % B was operated from 0 to 20 min and quassin had a retention time of ca 16 min. The data were acquired and processed with Agilent Chemstation software. Pure quassin was used as standard for preparing a calibration curve with peak area versus known amount of quassin. Pure quassin was isolated from *Q. amara* wood chips. One kg of wood chips was extracted for 24 h in 2 L of Milli-Q water. The extract was filtered and the water adjusted to pH 3.7 with HCl. The extract was partitioned three times with equal volumes of EtOAc. The EtOAc fractions were combined and stored at −20 °C over night. Water was removed from the EtOAc by filtering off the ice. The EtOAc was evaporated to dryness in a Rotavapor at 35 °C. The sample was dissolved in 1 mL water and injected in 100-μL aliquots into the HPLC and fractions corresponding to quassin were collected. The solvent was evaporated to dryness in a SpeedVac concentrator at 35 °C yielding crystalline quassin of high purity. A known amount of crystalline quassin was dissolved in 80 % water and 20 % MeOH and further diluted to prepare a standard curve showing amount of quassin versus UV absorption at 254 nm.

The chemical identity of the peak corresponding to putative quassin was established by ultra-high performance liquid chromatography (UHPLC)-electrospray ionization (ESI)-linear trap quadropole (LTQ) orbitrap mass spectrometry (MS). This method was used to find out mass to charge ratio of the particles for determining the elemental composition of quassin. Comparison was made with library data of fragmentation of quassin. Tandem mass spectra were used to further verify the chemical structure of the compound corresponding to putative quassin (Odén, P. C. et al., ms in preparation).

### Data analysis

The basic statistical assumption made when sawfly populations were studied across orchards and years was that populations are orchard specific, meaning that sawflies do not fly and mate across orchards (our study orchards were situated at least 20 km apart). The phenological characteristics of individual populations are therefore longitudinally transferred along the time-line within orchards, from 1 year to another. The statistical consequence of this defines the population concept; phenological covariance carries on from one generation to another, causing phenological observations to be inherited within, but not across, orchards. With such non-symmetric covariance structures being typical in longitudinal experimental designs, they are typically solved with a mixed linear model (Fitzmaurice et al. 2010). In statistical terms, the objective of the *Q. amara* trial was to determine whether treatments A–C had an effect on the relative proportions of damaged fruit as compared with the untreated control. In addition, it was investigated whether the effects of treatment varied temporally from one damage check occasion to another. This is basically an ANOVA setup with two fixed nominal factors *Treatment* and *Occasion*, where the effect of *Treatment* within the levels of *Occasion* the two levels of first and second damage check, where the interactive factor may be interpreted as addressing the temporal stability (homogeneity) of eventual treatment effects. In the present design, the first damage check occasion serves as a baseline for comparison with the second damage check (with null hypothesis that they do not differ), which eliminates the cumulative effect where the fruit damaged at occasion 1 also is counted at occasion 2. Furthermore, the basic ANOVA setup assumes normality and homoscedasticity, whereof neither was met. Instead, the response (damaged fruit) was binomial, with each apple either accepted or rejected. When this binomial process was summarized and compared with the total amount of fruit, a proportion in the interval p = [0, 1] was theoretically possible. However, when this interval was logit-transformed onto the interval [−∞, +∞], the resulting transformation could be considered approximately normal. The basic empirical assumption of sawfly phenology being inherited within orchards also contradicts the normality assumption of independent observations. This problem was solved with a mixed linear model, where the nominal factor *Orchard_id* was considered as being random, which introduced the concept of covariance to all observations grouped per orchard. Since the within-orchard design encompasses all possible sources of within-orchard covariance, effects such as orchard-specific climate, weather, management strategy, and autocorrelation characteristics were automatically captured with *Orchard_id*. When the above assumptions were combined into one inferential design, a mixed ANOVA was obtained where the response was a logit transformation of the proportions of damaged fruits.

The above mixed design was also applied to the relation between trap catches and flowering stages. In this case, a Box-Cox transformation of primary trap-catch counts was compared with the flowering stages BBCH 59, 60, 65, 67, and 69 (pre-bloom, incipient bloom, full bloom, incipient post-bloom, and full post-bloom, respectively). BBCH was considered to be an ordinal factor, with its effect on trap-catches estimated and plotted for visual interpretation. All mixed equations were solved with restricted maximum-likelihood algorithms, implemented in the software package STATISTICA ver. 12 (Statsoft 2014).

## Results

### Evaluation of first trap catch model

Trap catches of apple sawfly were observed from the beginning of May to mid-June. On average, first emergence of the sawfly occurred at 169 ± 20 degree days from March 15, compared with 177 ± 10 degree days as predicted by Zijp and Blommers (1997). The average difference between observed and predicted emergence was one calendar day or 9 degree days and the maximum difference was nine calendar days or 39 degree days (Table 2). However, 5 % of accumulated flight occurred on average 3 days after predicted first flight and never before April 30 in the 3 years of this study (Table 2). The average number of calendar days from predicted first flight to full bloom was 14 (Table 2).

**Table 2:** First trap catch and 5 % accumulated catch (acc.) of apple sawfly (*Hoplocampa testudinea*) (2011–2013) in seven Swedish apple orchards and the difference in degree days (DD) and calendar days as compared with the temperature sum of Zijp and Blommers (1997). Averages and SD comprise all years and orchards

Orchard	First catch (DD)	First catch (date)	Diff.^*^ (DD)	Diff. (days)	5 % acc. catch (DD)	5 % acc. catch (date)
1	195	2011-05-01	18	3	206	2011-05-06
	175	2012-05-07	−2	0	180	2012-05-08
	153	2013-05-09	−24	−3	163	2013-05-10
2	190	2011-04-30	13	2	195	2011-05-02
	160	2012-05-03	−17	−4	176	2012-05-07
	149	2013-05-09	−28	−3	167	2013-05-11
3	151	2013-05-09	−26	−3	182	2013-05-13
4	209	2011-05-09	32	9	209	2011-05-09
	174	2012-05-10	−3	0	174	2012-05-17
	166	2013-05-13	−11	−2	166	2013-05-16
5	175	2011-05-06	−2	−0	175	2011-05-06
	138	2012-05-03	−39	−8	152	2012-05-07
	143	2013-05-13	−34	−4	164	2013-05-16
6	189	2012-05-10	12	1	225	2012-05-18
	176	2013-05-13	−1	0	204	2013-05-17
7	153	2012-05-02	−24	−5	187	2012-05-09
	169 (SD = 20)		−9 (SD = 20)	−1 (SD = 3)	183 (SD = 20)	

^*^Difference to Zijp and Blommers (1997) temperature sum of 177 (SD 10) degree days

### Relationship between trap catches and flowering stages

Trap catches in all orchards had a strong tendency (*F*(3, 155) = 2.574, *p*< 0.056) to decrease at full flowering (BBCH 65), and increase again after petal fall (BBCH 69).

### Relationship between tree phenology (BBCH), egg, and larval development

**Fig. 1:**
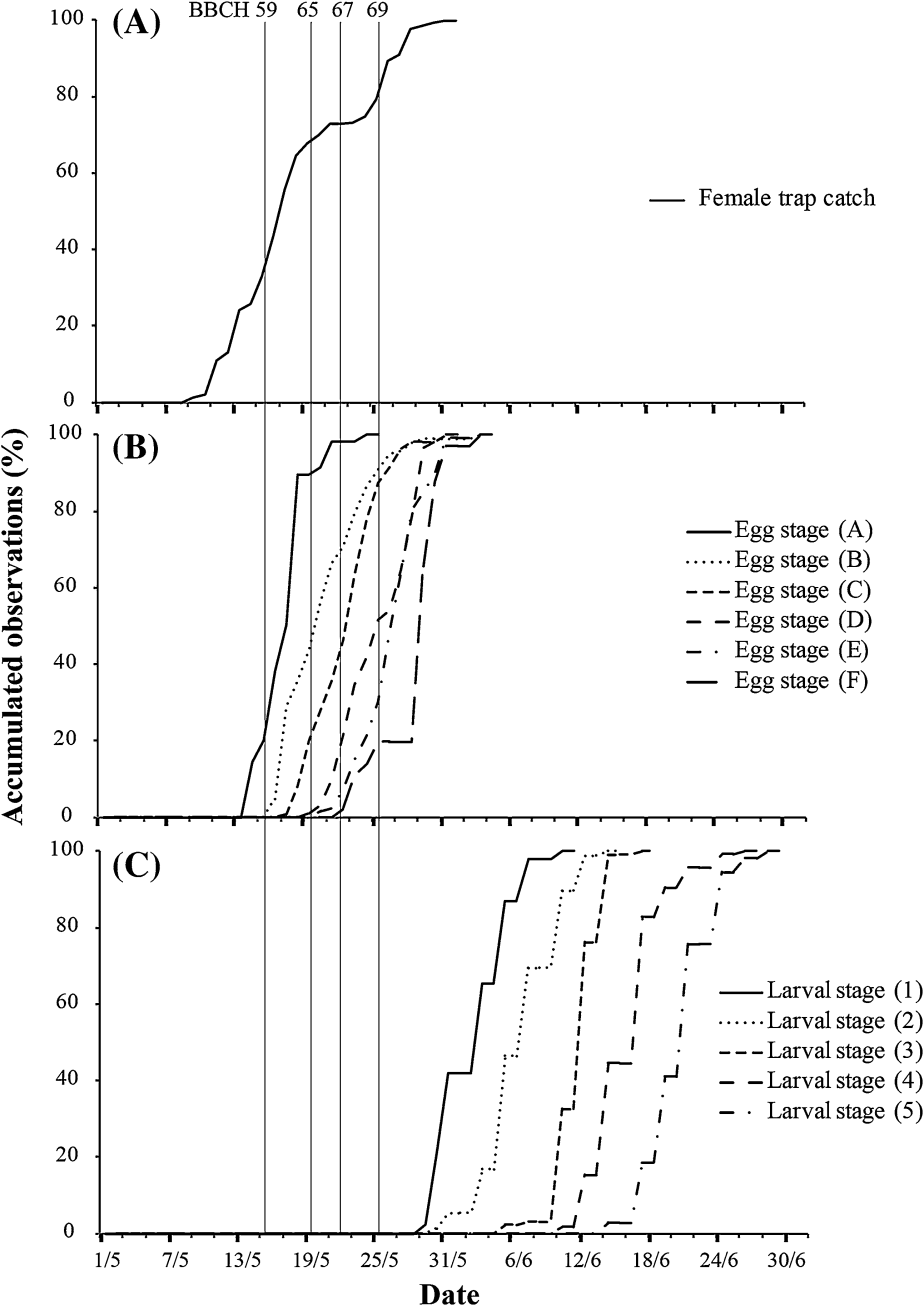
Apple sawfly (*Hoplocampa testudinea*) phenology on apple trees (BBCH scale as vertical lines) based on accumulated female trap captures (**a**), egg development (**b**), and larval development (**c**) (Sweden, 2013)

The first eggs were found 5–8 days after female sawflies emerged (Fig. 1). Prior to BBCH 65, approximately 85 % of all eggs were deposited but only 60 % of the total amount of female sawfly trap catches were recorded (Fig. 1). The period between first and last egg found for each respective egg stage lasted approximately 10 days, with a total egg stage period (dsA-dsF) of approximately 3 weeks (Fig. 1). The first sawfly larvae observed on apples appeared approximately 2 weeks after first appearance of the eggs (Fig. 1). The larval stage lasted approximately 30 days, with the period between first and last larva found for each larval stage lasting 10–15 days (Fig. 1). The average time from female flight to larval stage five was 32 days.

**Fig. 2:**
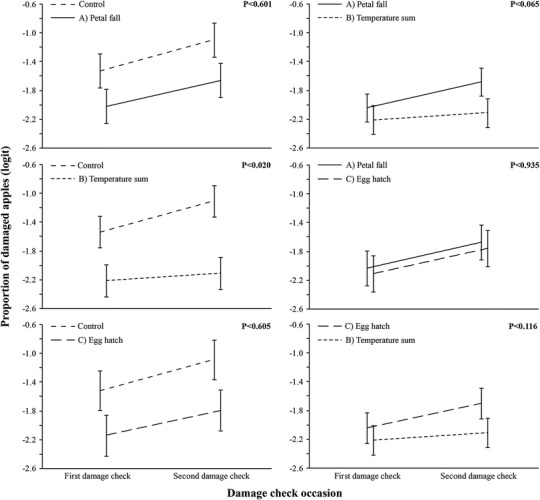
Apple sawfly (*Hoplocampa testudinea*) damage (± 95 % CI) in Swedish apple orchards (mixed model ANOVA with logit proportion pairwise interactions) with no treatment (control) or following sprays of *Quassia amara* extract according to timings of petal-fall (**a**), a female sawfly trap capture temperature sum model (**b**), and egg hatch (**c**)

### Application timing and efficacy of *Quassia amara*

At the second damage check occasion, the lowest level of accumulated damage (1.3 %) was obtained with treatment (trt) B, where *Q. amara* extract was applied according to combined female trap catch number and temperature sum (Graf et al. 2001, 2002) (Table 3). In all treatments, the total accumulated amount of damaged fruit was below 3.3 % and significantly lower than the 8.1 % damage in the control (Control-trt A, *F*(1, 283) = 16.99, *p*< 0.001; Control-trt B, *F*(1, 284) = 124.05, *p*< 0.001; Control-trt C, *F*(1, 243) = 49.58, *p*< 0.001, Table 3). Treatment B also showed lower damage levels than both Treatment A (*F*(1, 283) = 16.99, *p*< 0.001) and C (*F*(1, 195) = 11.34, *p*< 0.001) (Table 3), while the latter two did not differ significantly (*F*(1, 243) = 0.75, *p*< 0.388) (Table 3). Treatment B showed a significant interaction with the control (*F*(1, 282) = 5.46, *p*< 0.02, Fig. 2), which had increased damage levels at the second damage check. It did, however, not differ significantly from application at petal-fall (Treatment A; *F*(1, 282) = 3.44, *p*< 0.065) or at egg hatch (Treatment C; *F*(1, 194) = 2.49, *p*< 0.116) (Fig. 2), even though an increase in the mean damage was also observed for these treatments. With a maximum-likelihood algorithm used to estimate the effects of treatment, the latter two probability values may be interpreted as rather strong tendencies.

**Table 3:** Apple sawfly (*Hoplocampa testudinea*) damage in Swedish apple orchards following sprays of *Quassia amara* extract according to timings of petal-fall (**a**), a female sawfly trap capture temperature sum model (**b**), or egg hatch (**c**). Percent damage means followed by the same letter are not statistically different (alpha = 0.05)

Treatment	Year	Orchard	Application dates	First damage check	Second damage check	Average damage^*^(%)
A	2011	1	2011-05-19	2011-05-30	2011-06-15	2.7a
	2012	1	2012-05-24	2012-06-07	2012-06-20	
	2013	1	2013-05-23	2013-06-06	2013-06-19	
	2013	2	2013-05-23	2013-06-06	2013.06.19	
	2013	3	2013-05-23	2013-06-06	2013-06-19	
B	2013	1	2013-05-25	2013-06-06	2013-06-19	1.3b
	2013	2	2013-05-26	2013-06-06	2013-06-19	
	2013	3	2013-05-26	2013-06-06	2013-06-19	
C	2013	2	2013-05-29	2013-06-06	2013-06-19	3.3a
	2013	3	2013-05-29	2013-06-06	2013-06-19	
Control			All above dates			8.1c

^*^Average damage over years, orchards and both damage check occasions

## Discussion and Conclusion

### Evaluation of first trap catch model

After hibernating in the soil, the apple sawfly emerges in spring at a time primarily depending on soil/air temperature (Graf et al. 1996a, b; Zijp and Blommers 1997). Using the temperature sum construct proposed by (Zijp and Blommers 1997), we were able to estimate the timing of sawfly emergence with an average error of 1 day early and a maximum error of 9 days early. This relatively large deviation between studies may be explained by differences in population biology (Graf et al. 1996b) and regional differences as compared with the single Netherland field site used by Zijp and Blommers (1997). This deviation is acceptable since catching the first emerging individuals is not crucial for the purpose of mass trapping and monitoring peak flight. With the exception of one orchard (Table 2), five percent of accumulated flight occurred at average 3 days after the predicted first flight. Hence, to minimize the time during which traps are exposed and clogged with other insects, traps for monitoring or mass trapping could be placed according to Zijp and Blommers (1997) if the recommended safety margin of 157 degree days is used. First trap-catch occurred at average 14 days before full bloom (BBCH 65) and never before April 30. If temperature sums are not accessible then April 30 may be a simpler estimation for trap placement than the commonly recommended 10 days prior to bloom.

### Relation between trap catches and flowering stages

At several combinations of sites and years, a double trap-catch peak was observed. The strong tendency of decreasing adult densities observed between peaks occurred during full bloom. Since white sticky traps are designed for visual attraction (Wildbolz and Staub 1986), we interpret the trap catch decrease as being caused by competition from apple blossoms, as previously reported by (Haalboom 1983). This introduces an important bias to sawfly trap catches, where the seemingly interrupted flight curve probably reflects consistent adult activity.

### Relation between tree phenology (BBCH), egg, and larval development

Chaboussou (1961) observed that sawflies predominantly lay their eggs at BBCH 60–64 (Meier et al. 1994). This was confirmed to some extent in the present study, where more than 85 % of eggs were found prior to full bloom (BBCH 65). However, our observations regarding trapped adult females contradicted this, with only 60 % caught prior to full bloom (BBCH 65). This indicates that trap catches are not representative of egg deposition after BBCH 64. This inconsistency might explain the problems with finding a significant correlation between total trap-catch and fruit damage reported in previous studies (Graf et al. 1996c; Wildbolz and Staub 1986; Coli et al. 1985). In our orchards, the first sawfly larva was observed approximately 14 days after the first trap catch, which confirms the findings by Miles (1932).

Additionally, our observations indicate that the average time from peak flight to the final larval stage is 32 calendar days. This information could be used to calculate the timing of entomopathogenic fungi application. Previous studies have shown that entomopathogenic soil fungi (Jaworska 1992) and nematodes (Vincent and Bélair 1992) can contribute to decreasing sawfly populations. Therefore, in a future integrated approach, commercially available products of entomopathogenic fungi and/or nematodes could be applied just before sawfly larvae enter the soil.

### Application timing and efficacy of *Quassia amara*

Based on the above findings, we suggest that unless the aim is mass trapping, white sticky traps should be used primarily to measure the trap-catch peak of female sawflies prior to bloom, in order to help forecast the correct timing of *Q. amara* application. This suggestion is based on our *Q. amara* experiments, where the best effect (1.3 % fruit damage) was achieved with timing calculated as a combination of observed trap-catch peak of pre-bloom adult females and the temperature sums construct (Graf et al. 2001, 2002).

All three *Q. amara* application timings decreased the amount of damaged fruit as compared with the unsprayed control. The petal-fall treatment (A) was applied approximately 3 days earlier than the temperature sum treatment (B), i.e., a few days before the first larvae were found and while a majority of eggs were still not in the last stage. The egg-hatch treatment (C) was applied approximately 3 days after the temperature sum treatment, when the majority of the eggs had reached their final developmental stage. However, due to the difficulty in exact identification of this stage, a number of eggs could have hatched rather rapidly within the following hours and the corresponding neonate larvae could have entered the fruit before the insecticide spray. A quantitative study of *Q. amara* persistence in the field is lacking. However, the extract has been estimated to be active only over a short period of 4–6 days (Wijnen et al. 1994; Zijp and Blommers 2002a). It is, therefore, of primary importance to apply the extract during egg hatch, i.e., earlier than larval penetration McIndoo and Sievers 1917; Kienzle et al. 2002). In this study, the temperature sum application 3 days prior to peak egg hatch may have targeted a larger proportion of hatching eggs than application at the actual peak. For optimal application of insecticides with short persistency, there is a need for sufficient knowledge regarding the biology and phenology of the target insect. Adding a wetting agent can improve the effect of *Q. amara* treatment (Kienzle et al. 2006; McIndoo and Sievers 1917) but the improvement is not consistent (Paaske 2013). The results of the present study provide the possibility to use internationally proposed temperature sum constructs to create an application model for the Swedish climate. Growers unable to invest in weather stations for local temperature sum calculations could still use petal-fall to time their *Q. amara* application. However, in that case application should not be at 50 % petal fall (BBCH 67), but at the end of observed petal fall (BBCH 69).

A 4.8–6.8 % reduction in harvest results in a loss of 600–1600 euros/ha in Swedish organic production depending on the productivity of the cultivar. Furthermore, a *Q. amara* application prevents a gradual increase of the orchard population over the years. *Q. amara* is available both as an extract and as inexpensive wood chips for separate extraction, where the latter has been used in Sweden. Swedish growers use *Q. amara* to control both the apple sawfly and the rosy apple aphid simultaneously, which further increases its economic viability. At the moment, a formal registration process for *Q. amara* within the EU is underway.

The results from our study confirmed and clarified apple sawfly flight pattern, egg laying, and larval activity. It also showed that results from international studies on apple sawfly phenology can be used in Sweden as an effective tool to determine application timing. However, the findings should be validated with further studies in other regions and years.
